# Grip Force and 3D Push-Pull Force Estimation Based on sEMG and GRNN

**DOI:** 10.3389/fnins.2017.00343

**Published:** 2017-06-30

**Authors:** Changcheng Wu, Hong Zeng, Aiguo Song, Baoguo Xu

**Affiliations:** ^1^School of Instrument Science and Engineering, Southeast UniversityNanjing, China; ^2^College of Automation Engineering, Nanjing University of Aeronautics and AstronauticsNanjing, China

**Keywords:** grip force, 3D push-pull force, force estimation, EMG, GRNN

## Abstract

The estimation of the grip force and the 3D push-pull force (push and pull force in the three dimension space) from the electromyogram (EMG) signal is of great importance in the dexterous control of the EMG prosthetic hand. In this paper, an action force estimation method which is based on the eight channels of the surface EMG (sEMG) and the Generalized Regression Neural Network (GRNN) is proposed to meet the requirements of the force control of the intelligent EMG prosthetic hand. Firstly, the experimental platform, the acquisition of the sEMG, the feature extraction of the sEMG and the construction of GRNN are described. Then, the multi-channels of the sEMG when the hand is moving are captured by the EMG sensors attached on eight different positions of the arm skin surface. Meanwhile, a grip force sensor and a three dimension force sensor are adopted to measure the output force of the human's hand. The characteristic matrix of the sEMG and the force signals are used to construct the GRNN. The mean absolute value and the root mean square of the estimation errors, the correlation coefficients between the actual force and the estimated force are employed to assess the accuracy of the estimation. Analysis of variance (ANOVA) is also employed to test the difference of the force estimation. The experiments are implemented to verify the effectiveness of the proposed estimation method and the results show that the output force of the human's hand can be correctly estimated by using sEMG and GRNN method.

## Introduction

**Figure 1:**
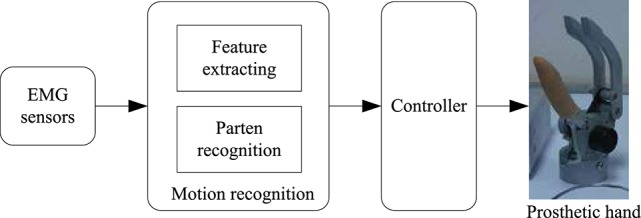
A typical control mode of the EMG prosthetic hand.

Prosthetic hand is a kind of human-machine interface. The upper limb amputees can recover some hand function with the help of the prosthetic hand. In recent years, many kinds of prosthetic hands have been investigated to meet the requirements of the amputees (Davidson, 2002; Zaini and Ahmad, 2011; Maat et al., 2017). Among these hands, the prosthetic hand based on the EMG has received lots of attention due to its simple operation and that it is in accordance with the operation habits of the natural hand. Figure 1 shows a typical block diagram of the EMG prosthetic hand. Firstly, the EMG signals are captured from the amputee's remaining arm by the EMG sensors on the skin surface. Then, the motion recognition unit outputs the user's motion intention based on analysis of the captured EMG signals. In the motion recognition unit, the features extracted from EMG signals are used to identify the user's motion intention. The controller sends the control commands to the prosthetic hand according to the user's motion intention which has been recognized in the motion recognition unit. In such manner, the prosthetic hand can be controlled by the amputee's EMG signals. The motion recognition unit plays an important role in this control mode. Whether the prosthetic hand movement is in line with the user's intention is directly decided by the motion recognition unit. As a matter of fact, many scholars have made a lot of investigations in this area (Zhang et al., 2013; Xie et al., 2015; Hofmann et al., 2016; Segil et al., 2016).

The hand movement is described by type, speed, force, and amplitude of the action, and etc. And all these are important in the control of the prosthetic hand, especially type and force. Therefore, the main tasks of the motion recognition are type classification and force estimation of the action.

In the process of type classification of the action, plenty of research work has been done in the past decades (Li et al., 2010; Hioki and Kawasaki, 2012; Ngeo et al., 2013; He et al., 2015; Pan et al., 2015). Nishikawa et al. used two channels of the EMG signals and the real-time learning method to discriminate a maximum 10 forearm motions including 4 wrist motions and 6 hand motions (Nishikawa et al., 1999). Ten different features extracted from four channels of the EMG signals are used to classify the eight hand motions in Al Omari et al. (2014). And the authors reported that an accuracy of 94% was achieved by using LDA (Linear Discriminant Analysis) based on the combination of wavelength, Willson amplitude, and root mean square. Wu proposed an EMG self-learning recognition method to reduce the effects of the individual difference on the EMG motion recognition (Wu et al., 2015). Ju and Liu used multiple sensors to analyze the human hand motions and reported that there exist significant relationships between the muscle signals and the finger trajectories as well as between the muscle signals and the contact force (Ju and Liu, 2014). To improve the performance of the EMG prosthetic hand, some scholars attempted to obtain the continuous motor variables such as the limb positions and force of the action. Ngeo used a multi-output convolved Gaussian Process to estimate the finger joint kinematics from the EMG signals (Ngeo et al., 2014).

The other part of the hand movement intention, force of hand movement (grip force, push force, pull force, etc.), is also important in the control of the prosthetic hand, especially in dexterous manipulation. There are mainly two kinds of force of the action output from the human hand. One is the grip force and the other is the push force and pull force in three dimensional space (we call it 3D force). In the process of grip force estimation, the most common method is detecting the strength of the muscle contraction from the EMG signals and then mapping the measured strength to the expecting grip force. During the bilateral grasping task, Kamavuako et al. investigated the use of the EMG features in order to estimate the grip force on the ipsilateral and contralateral hand (Kamavuako et al., 2012). And the author also reported that one channel of EMG signals measured from the flexor digitorum profundus can be used to represent the grip force within the entire range of force (Kamavuako et al., 2013). Castellini et al. used five channels of the EMG signals and the SVM (Support Vector Machine) to achieve the grip force estimation and their estimation error is <7% (Castellini et al., 2009). In 3D force estimation, Nielsen et al. used the artificial neural network to estimate the isometric force in multiple degrees of freedom from the wrist (Nielsen et al., 2011). Hashemi et al. researched the EMG based on force estimation in dynamic contractions by using the parallel cascade identification modeling (Hashemi et al., 2012, 2015).

Although, force estimation of the action is also important in the control of the prosthetic hand, the amount of the research work in this area is apparently less than the work in type classification of the action. Actually, not only the grip force but also the 3D force plays an important role in the control of the dexterous prosthetic hand. For the prosthetic hand with movable wrist, the 3D push/pull force is important in the process of moving objects. The estimate of 3D push/pull force can be used to control the rotation of the wrist.

In this paper, we present a force estimation method for the purpose of estimating the force of hand movement comprehensively. Firstly, we set up the experimental platform which is used to capture the EMG signals and force of the action. The eight channels of the EMG signals are captured from the eight different positions on the arm skin surface. The grip force and the 3D force are captured by a grip force sensor and a three dimensional force sensor, respectively. Secondly, the time domain feature extraction method is employed to extract the features from the captured EMG signals. Then, the generalized regression neural network (GRNN) is employed to estimate force of the action which includes the grip force and the 3D force, by using the extracted EMG features and the captured force of action. At last, experiments are implemented to verify the effectiveness of the proposed force estimation method of the action.

The rest of the paper is organized as follows. Section “Experimental Platform” introduces the experimental platform in detail. The detailed process of force estimation of the action is described in Section “Force Estimation of Hand Movement.” The experiments are implemented in Section “Experiment and Result,” and the Conclusions are in Section “Conclusion.”

## Experimental platform

**Figure 2:**
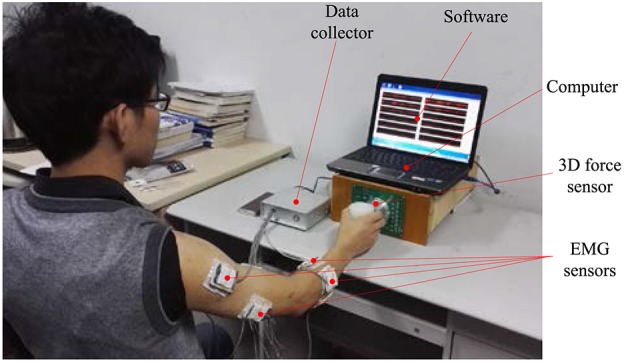
The experimental platform.

An experiment platform as shown in Figure 2 is set up in this section. The experimental platform consists of several EMG sensors, a three dimensional force sensor, a data collector, a computer, and the application software.

The EMG sensors and the 3D force sensor are respectively used to capture the surface EMG signals generated on the arm skin surface and the force signals output by the hand while the hand is moving and grasping. The EMG signals and the force signals are digitalized in the data collector. The data collector has a USB interface via which the data can be transmitted between the data collector and computer.

### EMG sensor

**Figure 3:**
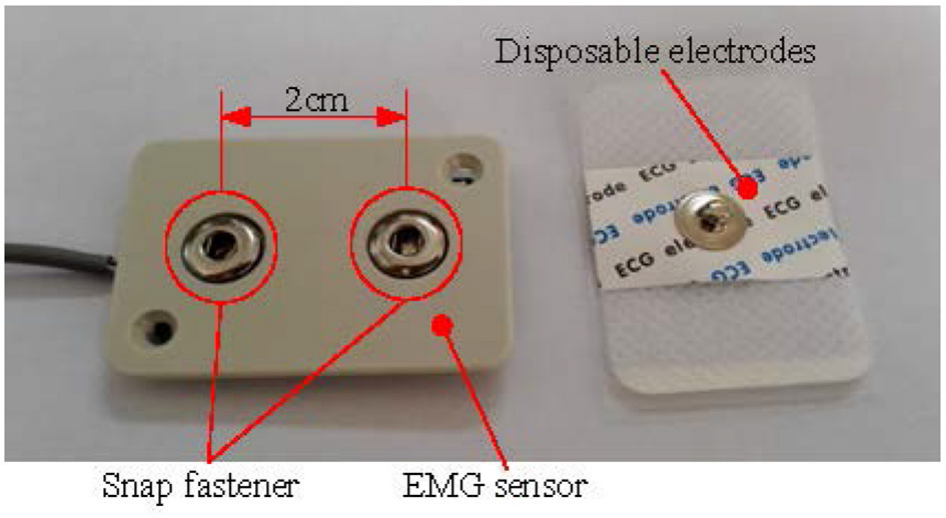
The EMG sensor and disposable electrode.

The EMG signal is a kind of weak signal. To meet the requirements of EMG signal measurement, we design a surface EMG sensor shown in Figure 3. The sensor's pass-band is 10–500 Hz and the voltage gain is about 1,000. The disposable moisture Ag/AgCl electrodes are used in the sensor. There are two snap-fasteners in the EMG sensor. The distance between these two snap-fasteners is 2 cm. And the snap-fastener is made of conductive metal. There is also a snap-fastener in the disposable electrode. The snap-fastener in the disposable electrode should be buckled into the snap-fastener in the EMG sensor.

**Figure 4:**
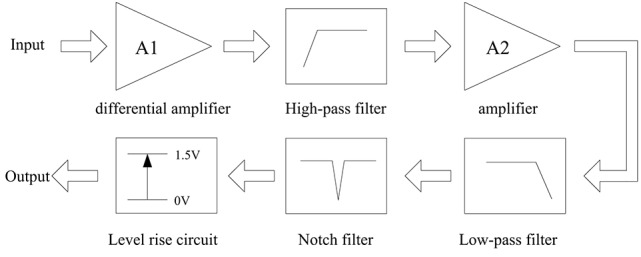
The structure diagram of the conditioning circuit.

**Figure 5:**
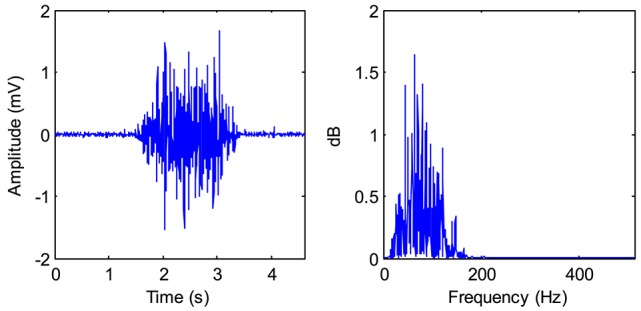
A typical EMG signal and its power spectrum.

There is a conditioning circuit in the EMG sensor. The structure diagram of the conditioning circuit is shown in Figure 4. The voltage gains of the two amplifiers, A1 and A2, are set as 15 and 40, respectively. The high-pass filter (cut-off frequency: 10 Hz) is used to remove the direct current component from the signal. The low-pass filter (cut-off frequency: 500 Hz) is used to eliminate the high-frequency noises. The voltage gains of these two filters are all about 1.3. The notch filter is used to reduce the 50 Hz power-line interference. Since the input range of the data collector is from 0 to 3 V, we design the level-rising circuit to make the voltage of the signals >0 V. Figure 5 shows a typical signal measured by the EMG sensor.

### Grip force sensor

**Figure 6:**
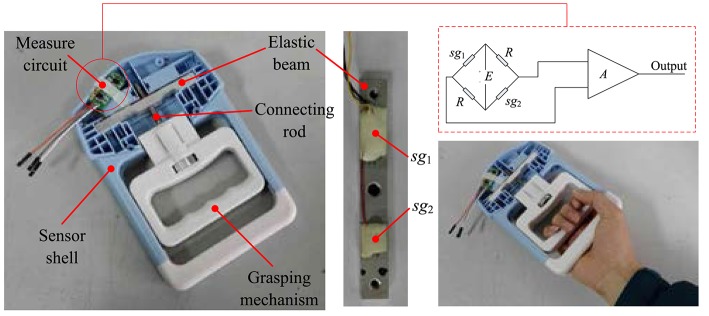
The grip force sensor and its manipulation sketch.

Figure 6 shows the grip force sensor and its manipulation sketch. The grip force sensor mainly contains a grasping mechanism (GM), an elastic beam, a signal conditioning circuit, and a sensor shell. There is a connecting rod between the elastic beam and the grasping mechanism. The elastic beam is fixed in the sensor shell. Grasping the GM with force will lead to the deformation of the elastic beam. There are two strain gauges, sg_1_ and sg_2_, attached on the surface of the elastic beam. These two strain gauges are used to measure the deformation of the elastic beam. According to the knowledge of the mechanics of the material, we know that there exists a relationship between the deformation of the beam and the force applied to the beam. Therefore, we can measure the grip force by detecting the deformation of the strain gauges. The widely used measurement circuit, Wheatstone bridge, is employed to measure the deformation of the strain gauges. And the output signals of the Wheatstone bridge are amplified by a differential amplifier.

The calibration is necessary for the reason that the relationship between the force applied to the elastic beam and the deformation of the beam is non-linear and there are some other factors which may influence the measurement accuracy. After the calibration experiment, the accuracy of the designed grip force sensor is 0.1N.

### 3D force sensor

**Figure 7:**
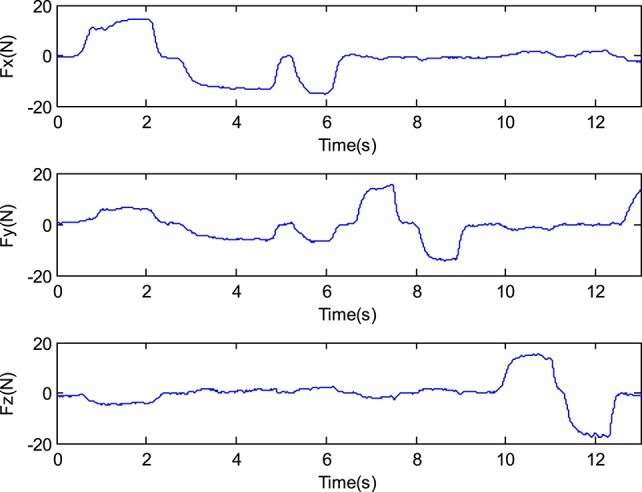
The force signals measured by the 3D force sensor.

A three dimension (3D) force sensor (measurement range: −30N ~30 N, accuracy: 2%FS), which is designed by the Robot Sensor and Control Lab in Southeast University, is introduced to measure the force signal (Ma and Song, 2012; Ma et al., 2013). Figure 7 shows the signals measured by the 3D force sensor while the force is applied to the sensor optionally. The value >0 indicates that the direction of the force is in forward direction, and the value <0 indicates that the direction of force is in reverse direction.

**Figure 8:**
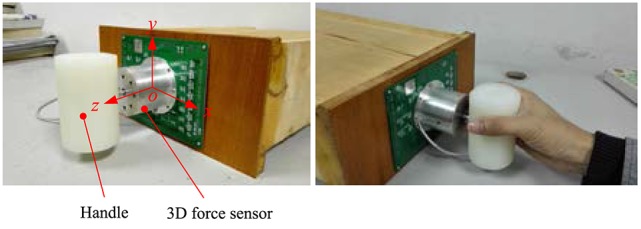
The 3D force sensor and its manipulation sketch.

As shown in Figure 8, the 3D force sensor is fixed on a vertical placed flat. And a cylindrical handle is installed at the end of the sensor. Holding the handle, human hand can apply force to the sensor in different directions.

### Data collector

Analogy signals captured by the sensors can be digitalized by the data collector. The designed data collector can realize analogy signal sampling in 16 channels simultaneously. The AD (analogy to digital) converters in the data collector are 10 bit. The sampling rate of the data collector can be set from 200 to 2000 Hz per channel. The voltage range of the input signals is from 0 to 3 V. The data collector has a USB interface through which data can be exchanged between the data collector and computer.

### Application software

The application software running on the computer is used to display the EMG signal waves and force signal waves in real time. And the data of these signals can be recorded when needed. In addition, the application software has the function of changing the data sampling rate by means of sending commands to the data collector.

## Force estimation of hand movement

**Figure 9:**
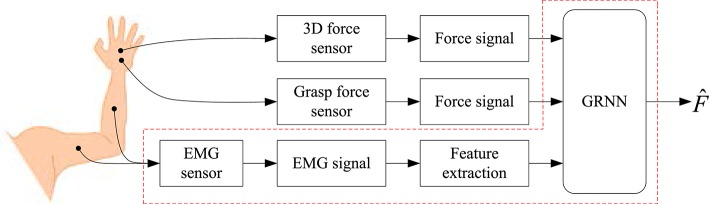
The diagram of the force estimation.

The relation between force and EMG signal is varied with many factors, such as force value, speed, path, etc. (Orizio et al., 2010; Kamavuako and Rosenvang, 2012). In order to study the relationship between the EMG signals and the force signals output by the hand and to estimate the force based on the EMG signal, we designed the signal processing diagram as shown in Figure 9.

Since the EMG signal is a kind of complex non-linear signal and the hand movement is completed by the cooperation of many muscles, it is difficult to describe the relationship between the EMG signals and the force output by the hand by using the linear model.

Under the assumption that there is a non-linear function, ϕ, which can describe the relationship between the EMG signals and the force output by the hand, the force output by the hand can be estimated by measuring the EMG signals on the arm skin surface.

(1) F=ϕ(X)

where, ϕ is the non-linear function, *X* are the EMG signals measured from the arm skin surface, *F* is the force output by the hand.

In this paper, the GRNN is employed to describe the non-linear relationship between the EMG signals and the force for its good non-linear mapping capability and high degree of parallel processing information capacity.

### EMG signal acquisition and feature extraction

**Figure 10:**
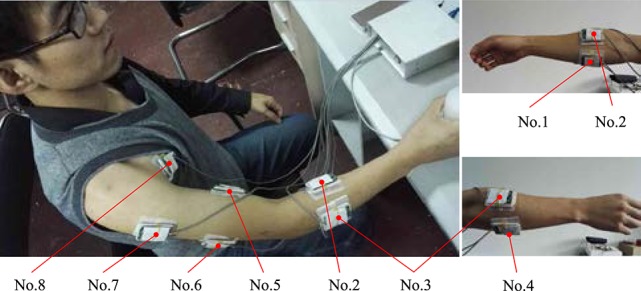
The distribution of the EMG sensors.

In this paper, eight different positions on the arm skin surface are selected to acquire the EMG signals. The distribution of the EMG sensors and the serial number of the EMG sensors are shown in Figure 10 and Table 1.

**Table 1:** The distribution location and the serial number of the EMG sensors.

**EMG sensor no**.	**Measuring site**
1	Lateral brachial wrist flexo
2	Brachioradialis
3	Extensor digitorum
4	Extensor carpi ulnaris muscle
5	Biceps
6	Triceps
7	Deltoid
8	Shoulder capsulorrhaphy

EMG signal captured by the data collector is a time series signal which can describe the characteristics of the hand movement after necessary preprocessing and feature extraction.

The feature extraction method of EMG signals is usually the time domain method, frequency domain method, and time-frequency domain method. The time domain method has the advantages of less computation comparing with the other two methods. The usual time domain feature extraction methods are mean absolute value (MAV), variance (VAR), zeros crossings (ZC), and Willison Amplitude (WA). Table 2 shows the mathematical equation of each introduced feature. All features are extracted by using Matlab.

**Table 2:** The EMG features in time domain and mathematical equation.

**Feature**	**Mathematical equation**
MAV	$MAV_{i}=\frac{1}{N}\overset{i}{\underset{j=i-N+1}{\sum }}|x_{j}|$
VAR	$VAR_{i}=\frac{1}{N}\overset{i}{\underset{j=i-N+1}{\sum }}x_{j}^{2}$
ZC	$ZC_{i}=\overset{i}{\underset{j=i-N+1}{\sum }}sgn\left(x_{j}x_{j-1}\right)$
	$sgn(x)=\{\begin{array}{cc} 1, & x>0\\ 0, & x\leq 0 \end{array}$
WA	$WA_{i}=\overset{i}{\underset{j=i-N+2}{\sum }}f\left(x_{i}-x_{i-1}\right)$
	$f(x)=\{\begin{array}{c} 1,\;|x|>threadhold\\ 0,\;others\; \end{array}$

*Where, x_j_ is the jth sample data, N is the length of the sliding window*.

**Figure 11:**
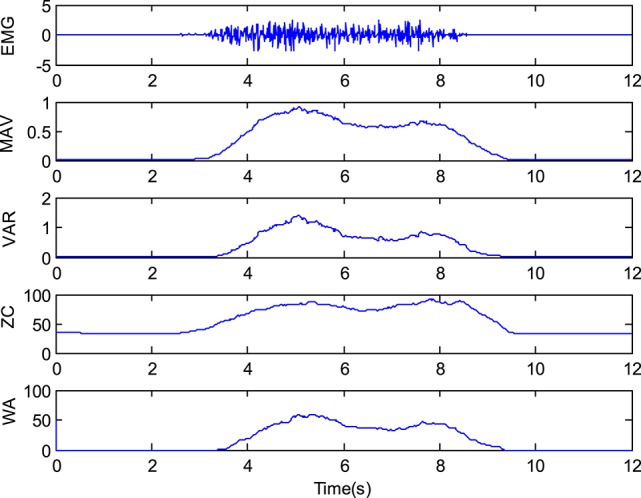
A typical EMG signal and its time domain features.

Figure 11 shows a typical EMG signal and its time domain feature.

### The generalized regression neural network

The GRNN was proposed by Donald F. Specht in 1991. It is a kind of RBF (Radial Basis Function) neural network. It has the advantage of non-linear mapping, great robustness, and a high degree of parallel processing information capacity. The performance and the learning speed of the GRNN are better than the general neural network (Altiparmak et al., 2009).

**Figure 12:**
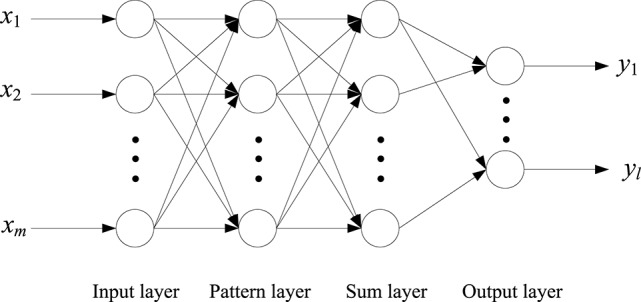
The structure of the GRNN.

The GRNN has a network structure of 4 layers as shown in Figure 12. These four layers are input layer, pattern layer, sum layer, and output layer, respectively (Altiparmak et al., 2009).

*X* = [*x*_1_, *x*_2_,…,*x*_*m*_]^*T*^ is the input vector of the GRNN, and *Y* = [*y*_1_, *y*_2_,…,*y*_*l*_]^*T*^ is the output of the GRNN. The number of the neuron in pattern layer is as the same as the number of the training samples. Each neuron in pattern layer corresponds to a training sample data. The transfer function of the *i*th neuron in pattern layer is:

(2) $$P_{i}=\exp[\frac{{\left(X-X_{i}\right)}^{T}(X-X_{i})}{2\sigma^{2}}],i=1,2,...,n$$

where, *X* is the input of the GRNN, *X*_*i*_ is the learning sample corresponding to *i*th neuron, σ is the smoothing parameter.

The neuron in sum layer can be divided into two classes. One class corresponds to Equation (3), and the number is only one. The other class corresponds to Equation (4).

(3) $$S_{D}=\sum_{i\;=\;1}^{n}P_{i}$$

(4) $$S_{Nj}=\sum_{i\;=\;1}^{n}y_{ij}P_{i},\;j=1,2,...,L$$

where, *y*_*ij*_ is *jth* element in *Y*_*i*_, *Y*_*i*_ is the *ith* output sample. *L* is the dimension of the output vector.

The output of the GRNN is defined as:

(5) $$y_{i}=\frac{S_{Ni}}{S_{D}}i=1,2,...,L$$

To improve the performance of the GRNN in our study, the samples should be distributed as uniformly as possible within a certain range.

### Force estimation of hand movement

In this paper, the GRNN is employed to study the relationship between the EMG signals and the force output by the hand. As shown in Figure 9, the force estimation can be divided into two steps. One is offline training of the GRNN, and the other is the online force estimation. In the prosthetic hand based on EMG, the GRNN is offline trained by collecting the EMG signals and the force signals. The trained GRNN is used to estimate force of the action in real time. It is that mapping the extracted EMG features into the force of hand movement by using the trained GRNN.

The software in the computer records the eight channels of the EMG signals (*X*), the grip force signals (*F*_*g*_), and the 3D force signals (*F*_3*D*_) in real time.

(6) $$X=\left[\begin{array}{c} X_{1}\\ X_{2}\\ \vdots \\ X_{n} \end{array}\right]=\left[\begin{array}{cccc} x_{11} & x_{12} & \cdots & x_{1m}\\ x_{21} & x_{22} & \cdots & x_{2m}\\ \vdots & \vdots & \vdots & \vdots \\ x_{n1} & x_{n2} & \cdots & x_{nm} \end{array}\right]$$

(7) $$F_{3D}=\left[\begin{array}{c} F_{x}\\ F_{y}\\ F_{z} \end{array}\right]=\left[\begin{array}{cccc} f_{x1} & f_{x2} & \cdots & f_{xm}\\ f_{y1} & f_{y2} & \cdots & f_{ym}\\ f_{z1} & f_{z2} & \cdots & f_{zm} \end{array}\right]$$

(8) $$F_{g}=\left[\begin{array}{cccc} f_{g1} & f_{g2} & \cdots & f_{gm} \end{array}\right]$$

where, *X*_1_ ~ *X*_8_ are the eight channels of the EMG signals captured by the eight EMG sensors, respectively, *x*_*ij*_ is the *jth* sample data captured by the *ith* EMG sensor. *F*_*x*_, *F*_*y*_, and *F*_*z*_ are the force signal in *x, y*, and *z* direction measured by the 3D force sensor, respectively; *f*_*xi*_, *f*_*yi*_, *f*_*zi*_ are the *ith* sample data in *x, y*, and *z* direction, respectively; *f*_*gi*_ is the *ith* sample data captured by the grip force sensor and *m* is the number of the sampling.

Time domain feature extraction methods described in Table 2 are used in the processing of the EMG signals. And we can get the feature matrixes: *XF*_*MAV*_, *XF*_*VAR*_, *XF*_*ZC*_, and *XF*_*WA*_.

(9) $$XF_{MAV}=\left[\begin{array}{c} X_{1MAV}\\ X_{2MAV}\\ \vdots \\ X_{8MAV} \end{array}\right]\;XF_{VAR}=\left[\begin{array}{c} X_{1VAR}\\ X_{2VAR}\\ \vdots \\ X_{8VAR} \end{array}\right]$$

(10) $$XF_{ZC}=\left[\begin{array}{c} X_{1ZC}\\ X_{2ZC}\\ \vdots \\ X_{8ZC} \end{array}\right]\;XF_{WA}=\left[\begin{array}{c} X_{1WA}\\ X_{2WA}\\ \vdots \\ X_{8WA} \end{array}\right]$$

Combining the feature matrixes and the force signals, we can get a new matrix *S*_*am*_.

(11) $$S_{am}=\left[\begin{array}{c} XF\\ F \end{array}\right]$$

where, *XF* is one of the *XF*_*MAV*_, *XF*_*VAR*_, *XF*_*ZC*_, and *XF*_*WA*_*. F* is one of the *F*_3*D*_ and *F*_*g*_.

In the offline training step of the GRNN, the *k*-fold cross validation is introduced to evaluate the performance of the GRNN.

1. The *S*_*am*_ is split into *k* equal-size groups of size *m/k*.
2. One of the groups is used as validation group in order to test the performance of the GRNN; the rest *k–*1 groups are used in training process.
3. The cross validation process is repeated *k* times with different selected groups with the mean accuracy.

In the *k*-fold cross validation method, *k* is set to two in the paper.

The mean absolute value of the estimation error (*MAVE*), the root mean square (*RMS*) of the estimation error and the correlation coefficient (ρ) between *F* and $\tilde{F}$ are introduced to evaluate the accuracy of the estimation results. Take the force in direction *x* for an example:

(12) $$MAVE_{x}=\frac{\sum_{i\;=\;1}^{N}abs({\tilde{f}}_{xi}-f_{xi})}{N}$$

(13) $$RMS_{x}=\sqrt{\frac{\sum_{i\;=\;1}^{N}{\left({\tilde{f}}_{xi}-f_{xi}\right)}^{2}}{N-1}}$$

(14) $$\rho_{{\tilde{f}}_{x},f_{x}}=\frac{\mathrm{cov}({\tilde{f}}_{x},f_{x})}{\sigma_{{\tilde{f}}_{x}}\sigma_{f_{x}}}=\frac{E[({\tilde{f}}_{x}-\mu_{{\tilde{f}}_{x}})(f_{x}-\mu_{f_{x}})]}{\sigma_{{\tilde{f}}_{x}}\sigma_{f_{x}}}$$

where, ${\tilde{f}}_{xi}$ is the estimate of force in direction *x*; *f*_*xi*_ is the force in direction *x* measured by the 3D force sensor; *N* is the length of the data.

A greater *MAVE* indicates that the estimation result is poorer. A greater *RMS* indicates that the estimation result has a larger fluctuation. And a greater ρ indicates that the estimated result has a higher similarity with the force measured by the force sensor.

In grip force estimation experiments, One-way ANOVA is conducted to compare the performance of different features In One-way ANOVA, the factor is EMG feature (MVA, VAR, ZC, WA). In 3D force estimation experiments, Two-way ANOVA is conducted. The factors of the Two-way ANOVA are force direction (*x, y, z*) and EMG feature (MVA, VAR, ZC, WA). Tukey comparison is performed when significance is detected. The significance level for all tests is set at 0.05.

## Experiment and result

To verify the effectiveness of the proposed method in this paper, the experiment was implemented.

Six right hand-dominant healthy subjects without any neuromuscular disorders (4 males, 2 females, and aged between 21 and 28) were chosen as the participants the experiment. And the participants were informed consent prior to study participation.

Before the experiment, the locations of the EMG sensors on the arm skin surface were cleaned by medical alcohol. Then, the electrodes were buckled into the EMG sensors and the EMG sensors were attached on the arm skin surface. The locations of the EMG sensors are shown in Table 1 and Figure 10.

During the experiments, the EMG signals and force signals of the action were displayed as waveforms in the computer screen and recorded by the software. The data sampling rate of the data collector was set as 1 kHz per-channel.

In the experiments, the data captured from different individuals are used to train the GRNN, respectively.

### The grip force estimation experiment

Firstly, the grip force estimation experiment is implemented. During the grip force estimation experiment, the subjects are asked to grasp the grip force sensor several times with different grip force. During the grasping, the subjects should not move their arms. The duration of the grasping is about 1–2 s. The maximum grip force is <30N.

**Figure 13:**
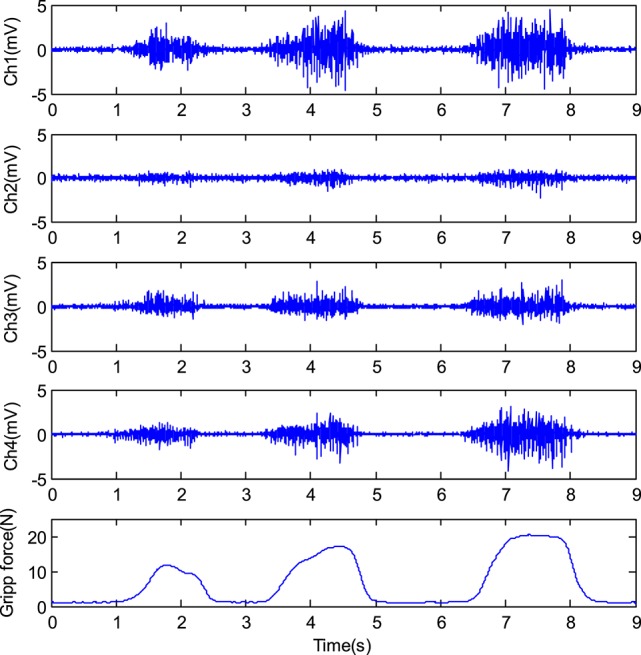
The EMG signals and the corresponding grip force.

In the experiment, four channels of the EMG signals were selected to estimate the grip force. Figure 13 shows the EMG signals and the grip force while the subject 1 was grasping the grip sensor three times continuously with different grip force.

Four kinds of EMG time domain features which were extracted from the same raw EMG signals were used to train the GRNN, respectively. And the trained GRNNs were used to estimate the grip force, respectively.

**Figure 14:**
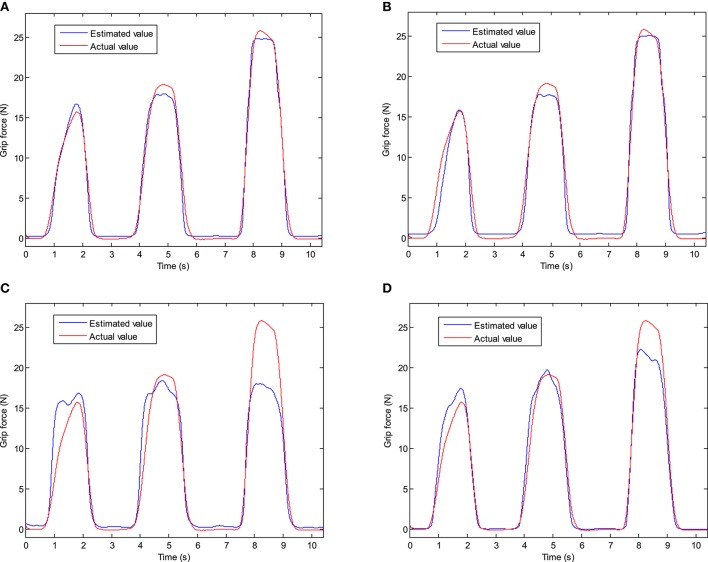
The experiment results of the grip force estimation. **(A)** Feature = MAV. **(B)** Feature = VAR. **(C)** Feature = ZC. **(D)** Feature = WA.

The experiment results of the subject 1 are shown in Figure 14. It shows that the results of the WAV and VAR are better than the results of ZC and WA. When the grip force increases from 0 to 8N, the results of all features show a good estimation effect. When the grip force is larger than 10N, the estimation accuracy of ZC and WA decreases, especially when the force is larger than 15N. In the results of ZC, the fluctuation may appear in the estimation results when the grip force is larger than 15N. And when the grip force is larger than 15N, the estimation accuracy is decline sharply in the result of ZC. When the grip force is larger than 20N, the estimation accuracy is also decline in the result of WA. In general, the estimate of force by using these four features and the actual force have the similar trend.

**Figure 15:**
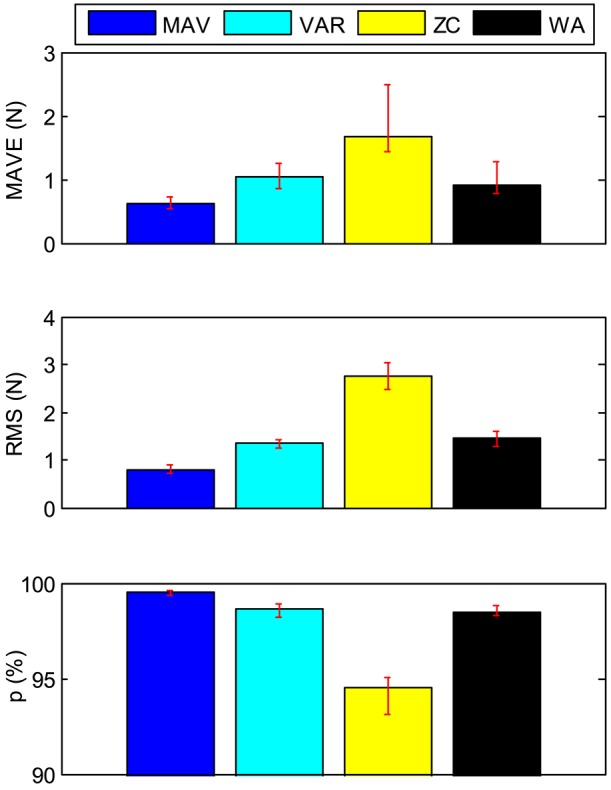
The statistical results of the grip force estimation experiments.

The results of all six subjects are shown in Table 3. The average values of the six subjects' results are calculated and shown in Table 4 and Figure 15. The variation range of the all subjects' results is also shown in the form of vertical red line, as shown in Figure 15. It shows that the result of MVA is better than the other features'. The result of ZC has a larger fluctuation than that of the other three features.

**Table 3:** The results of the grip force estimation experiments.

		**Grip force**		
		***MAVE* (*N*)**	***RMS* (*N*)**	**ρ (%)**
Subject 1	MAV	0.69	0.89	99.46
	VAR	1.12	1.36	98.75
	ZC	1.87	2.85	94.72
	WA	1.06	1.61	98.27
Subject 2	MAV	0.55	0.73	99.62
	VAR	1.07	1.36	98.64
	ZC	1.70	2.76	94.79
	WA	0.84	1.27	98.85
Subject 3	MAV	0.63	0.79	99.58
	VAR	1.03	1.28	98.91
	ZC	1.82	2.82	94.89
	WA	1.06	1.53	98.42
Subject 4	MAV	0.63	0.82	99.38
	VAR	1.10	1.41	98.20
	ZC	1.43	2.49	94.64
	WA	0.77	1.28	98.56
Subject 5	MAV	0.62	0.77	99.56
	VAR	1.12	1.40	98.51
	ZC	1.83	3.03	93.13
	WA	0.97	1.53	98.29
Subject 6	MAV	0.54	0.79	99.56
	VAR	0.85	1.24	98.89
	ZC	1.44	2.63	95.08
	WA	0.84	1.44	98.53

**Table 4:** The average results of the grip force estimation experiments across all the subjects.

	**Grip force**		
	***MAVE* (*N*)**	***RMS* (*N*)**	**ρ (%)**
MAV	0.61	0.80	99.53
VAR	1.05	1.34	98.65
ZC	1.68	2.76	94.54
WA	0.92	1.44	98.49

The results of One-way ANOVA and Tukey HSD are shown in Tables 5–8. Results in Table 5 indicate that there are significant differences (*p* < 0.05) among four features. The results of the Tukey HSD show that there are significant differences between each two features except VAR and WA. Four features can be divided into three subsets.

**Table 5:** The results of the One-way ANOVA.

		**Sum of squares**	***df***	**Mean square**	***F***	**Sig**.
MAVE	Between groups	3.646	3	1.215	70.554	0.000
	Within groups	0.345	20	0.017		
	Total	3.990	23			
RMS	Between groups	12.519	3	4.173	268.170	0.000
	Within groups	0.311	20	0.016		
	Total	12.831	23			
ρ	Between groups	88.753	3	29.584	188.823	0.000
	Within groups	3.134	20	0.157		
	Total	91.886	23			

**Table 6:** The results of the Tukey HSD for *MAVE*.

***MAVE***				
**Feature**	***N***	**Subset for alpha = 0.05**		
		**1**	**2**	**3**
MAV	6	0.6100		
WA	6		0.9233	
VAR	6		1.0483	
ZC	6			1.6817
Sig.		1.000	0.375	1.000

**Table 7:** The results of the Tukey HSD for *RMS*.

***RMS***				
**Feature**	***N***	**Subset for alpha = 0.05**		
		**1**	**2**	**3**
MAV	6	0.7983		
VAR	6		1.3417	
WA	6		1.4433	
ZC	6			2.7633
Sig.		1.000	0.507	1.000

**Table 8:** The results of the Tukey HSD for ρ.

**ρ**				
**Feature**	***N***	**Subset for alpha = 0.05**		
		**1**	**2**	**3**
ZC	6	94.5417		
WA	6		98.4867	
VAR	6		98.6500	
MAV	6			99.5267
Sig.		1.000	0.890	1.000

The above results indicate that the result of the WAV is better than the other three features' results by all three evaluation index. The result of the VAR and the result of the WA are similar. Among all the results, the ZC get the worst one. The results also show that all of these four features can realize the estimation of the grip force in an acceptable performance.

### The 3D force estimation experiment

The 3D force is the representative of the force output by the human hand in three-dimensional space, which can be measured by 3D force sensor. During the 3D force estimation experiment, the subjects were asked to grasp the handle which was set at the end of the 3D force sensor, and to apply force to the handle in *x, y*, and *z* direction. The duration of the subjects applying force to each direction is about 1 s. The maximum force is <20N.

**Figure 16:**
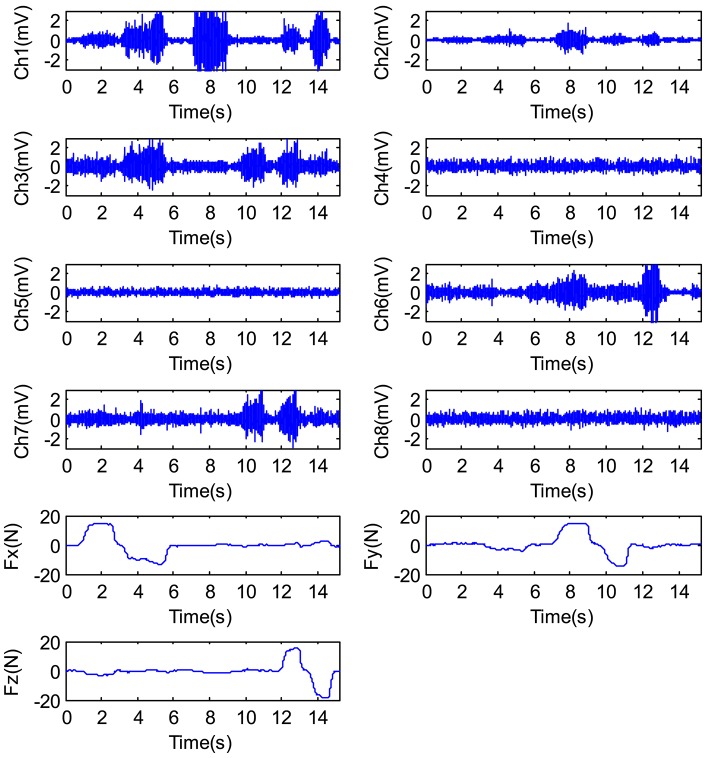
The EMG signals and force signals output by the hand.

In this experiment, eight channels of the EMG signals were selected to estimate the grip force. Figure 16 shows the EMG signals and the 3D force while subject 1 applied force to the 3D force sensor.

The same as the grip force estimation experiment, four kinds of EMG time domain features which were extracted from the same raw EMG signals were used to train the GRNN, respectively. And the trained GRNNs were used to estimate the 3D force, respectively.

**Figure 17:**
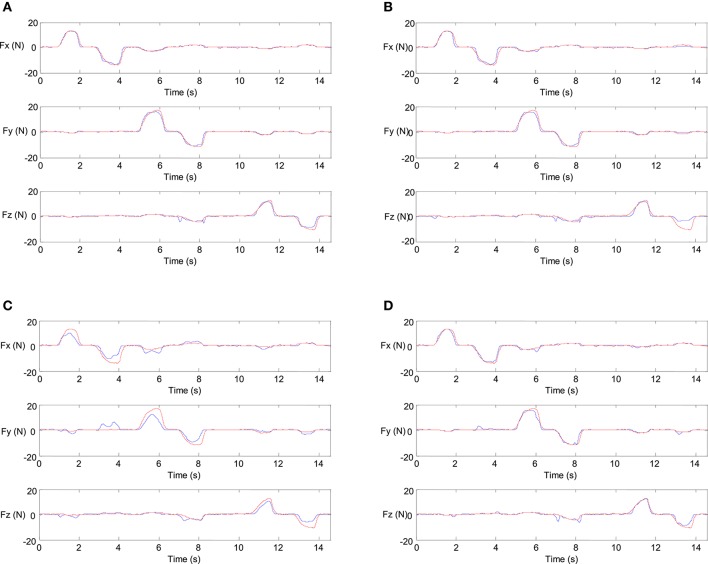
The experimental results of the 3D force estimation. **(A)** Feature = *WAV*. **(B)** Feature = *VAR*. **(C)** Feature = *ZC*. **(D)** Feature = *WA*.

The experimental results of the subject 1 are shown in Figure 17. In Figure 17, the red line represents the actual force and the blue line represents the estimated force. The result of WAV has the best performance among the results of all these four features. And the result of ZC is the worst. When applying force to *z*-direction, which means pushing the 3D force sensor, the estimation results are poor by using the features of VAR and ZC. On the whole, the 3D force estimation results are acceptable.

**Figure 18:**
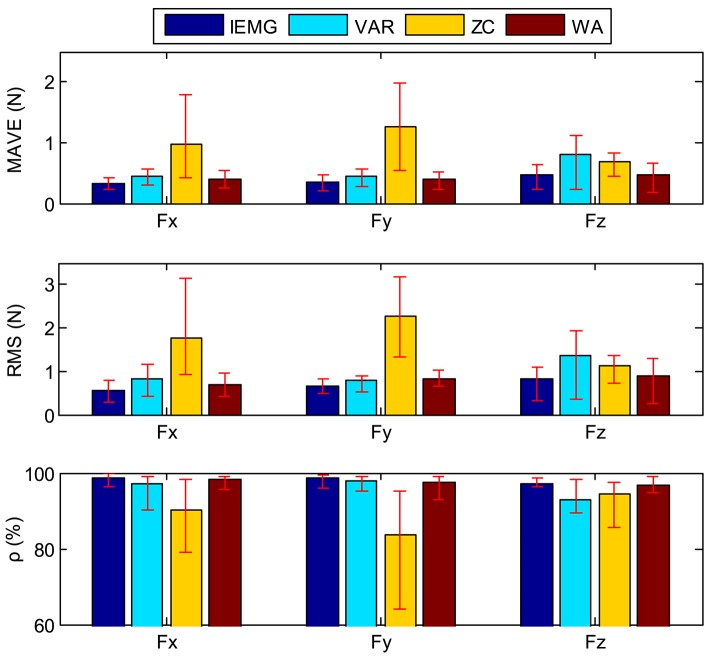
The statistical results of the 3D force estimation experiments.

The results from all six subjects are shown in Tables 9, 10. The average values and the variation range of the six subjects' results are shown in Figure 18 indicated by bar graph and vertical red line, respectively. It shows that the result of MVA is better than the other features'. The result of ZC has a larger fluctuation than that of the other three features. In the *z*-direction, the result of VAR also has a lager fluctuation. And in the *z*-direction, the result of VAR is worst by all three evaluation index.

**Table 9:** The results of the 3D force estimation experiments.

		***Fx***			***Fy***			***Fz***		
		***MAVE* (N)**	***RMS* (N)**	**ρ (%)**	***MAVE* (N)**	***RMS* (N)**	**ρ (%)**	***MAVE* (N)**	***RMS* (N)**	**ρ (%)**
Subject 1	*MAV*	0.42	0.70	99.13	0.44	0.78	99.05	0.22	0.32	96.47
	*VAR*	0.53	0.98	98.31	0.49	0.89	98.81	0.24	0.36	95.74
	*ZC*	1.78	3.15	86.56	1.98	3.19	86.34	0.45	0.71	85.51
	*WA*	0.48	0.76	99.02	0.50	1.02	98.45	0.18	0.26	97.68
Subject 2	*MAV*	0.38	0.68	98.44	0.46	0.78	99.06	0.34	0.50	98.65
	*VAR*	0.45	0.83	97.75	0.52	0.87	98.86	0.37	0.58	98.24
	*ZC*	1.18	2.03	86.65	1.66	2.75	89.59	0.61	1.07	95.70
	*WA*	0.43	0.74	98.24	0.51	1.02	98.44	0.28	0.44	99.03
Subject 3	*MAV*	0.28	0.48	96.33	0.47	0.83	98.95	0.60	1.04	97.01
	*VAR*	0.39	0.79	90.22	0.56	0.88	98.82	1.11	1.85	90.34
	*ZC*	0.42	1.08	79.14	1.02	1.86	95.28	0.69	1.11	97.54
	*WA*	0.30	0.54	95.56	0.45	0.82	98.99	0.64	1.22	95.80
Subject 4	*MAV*	0.22	0.29	99.55	0.33	0.64	98.55	0.64	1.11	96.48
	*VAR*	0.31	0.42	99.05	0.43	0.77	97.96	1.12	1.92	89.43
	*ZC*	0.51	0.93	96.26	1.09	2.07	86.10	0.77	1.18	96.97
	*WA*	0.26	0.41	99.15	0.34	0.71	98.24	0.66	1.31	94.98
Subject 5	*MAV*	0.28	0.44	99.63	0.21	0.48	96.05	0.54	0.98	97.13
	*VAR*	0.42	0.76	98.90	0.27	0.52	95.37	1.04	1.82	90.42
	*ZC*	0.69	1.16	98.29	0.54	1.32	64.15	0.80	1.32	95.73
	*WA*	0.36	0.64	99.19	0.24	0.65	92.87	0.56	1.12	96.28
Subject 6	*MAV*	0.41	0.79	98.87	0.26	0.48	99.26	0.50	0.89	97.63
	*VAR*	0.57	1.17	97.54	0.36	0.74	98.23	0.91	1.65	92.44
	*ZC*	1.29	2.21	94.03	1.35	2.44	80.13	0.82	1.35	95.51
	*WA*	0.53	0.95	98.39	0.35	0.76	98.16	0.50	0.98	97.06

**Table 10:** The average results of the 3D force estimation experiments across all the subjects.

	***Fx***			***Fy***			***Fz***		
	***MAVE* (*N*)**	***RMS* (*N*)**	**ρ (%)**	***MAVE* (*N*)**	***RMS* (*N*)**	**ρ (%)**	***MAVE* (*N*)**	***RMS* (*N*)**	**ρ (%)**
*MAV*	0.33	0.56	98.66	0.36	0.67	98.49	0.47	0.81	97.23
*VAR*	0.45	0.83	96.96	0.44	0.78	98.01	0.80	1.36	92.77
*ZC*	0.98	1.76	90.16	1.27	2.27	83.60	0.69	1.12	94.49
*WA*	0.39	0.67	98.26	0.40	0.83	97.53	0.47	0.89	96.81

The results of Two-way ANOVA and Tukey HSD are shown in Tables 11–13. The results of the Tukey HSD are shown in Tables 13–16. The results of Two-way ANOVA indicate that there are significant differences (*p* < 0.05) among four features. For all three evaluation index, the effects of force direction are not significant (*p* > 0.456). The results of Tukey HSD show that the effects of MAV, VAR, and WA are similar. Four features can be divided into two subsets.

**Table 11:** The results of the Two-way ANOVA for *MAVE*.

**Source**	**Type III Sum of squares**	***df***	**Mean square**	***F***	**Sig**.
Corrected model	4.099	5	0.820	9.471	0.000
Intercept	24.863	1	24.863	287.250	0.000
Force direction	0.093	2	0.047	0.538	0.586
EMG_Feature	4.006	3	1.335	15.426	0.000
Error	5.713	66	0.087		
Total	34.675	72			
Corrected total	9.811	71			

**Table 12:** The results of the Two-way ANOVA for *RMS*.

**Source**	**Type III sum of squares**	***df***	**Mean square**	***F***	**Sig**.
Corrected model	12.135	5	2.427	9.820	0.000
Intercept	78.730	1	78.730	318.566	0.000
Force direction	0.392	2	0.196	0.794	0.456
EMG_Feature	11.743	3	3.914	15.838	0.000
Error	16.311	66	0.247		
Total	107.177	72			
Corrected total	28.446	71			

**Table 13:** The results of the Two-way ANOVA for ρ.

**Source**	**Type III sum of squares**	***df***	**Mean square**	***F***	**Sig**.
Corrected model	893.973	5	178.795	7.522	0.000
Intercept	653,163.541	1	653,163.541	27,478.191	0.000
Force direction	31.084	2	15.542	0.654	0.523
EMG_Feature	862.888	3	287.629	12.100	0.000
Error	1568.837	66	23.770		
Total	655,626.351	72			
Corrected total	2462.809	71			

**Table 14:** The results of the Tukey HSD for *MAVE*.

**EMG_Feature**	***N***	**Subset**	
		**1**	**2**
MAV	18	0.3889	
WA	18	0.4206	
VAR	18	0.5606	
ZC	18		0.9806
Sig.		0.306	1.000

**Table 15:** The results of the Tukey HSD for *RMS*.

**EMG_Feature**	***N***	**Subset**	
		**1**	**2**
MAV	18	0.6783	
WA	18	0.7972	
VAR	18	0.9889	
ZC	18		1.7183
Sig.		0.249	1.000

**Table 16:** The results of the Tukey HSD for ρ.

**EMG_Feature**	***N***	**Subset**	
		**1**	**2**
ZC	18	89.4156	
VAR	18		95.9128
WA	18		97.5294
MAV	18		98.1244
Sig.		1.000	0.528

The above results indicate that all of these four features can achieve the estimation of the 3D force successfully. The result of MAV is best. But compare with VAR and WA, there is no significant difference. The result of ZC is worst.

## Conclusion

In order to meet the requirements of the dexterous control of the prosthetic hand, the paper proposes a force estimation method of hand movement based on the sEMG and GRNN. The estimated force of hand movement includes the grip force and the 3D force. An experimental platform is set up to measure the multi-channels of the sEMG signals, the grip force and the 3D push-pull force. Based on this platform, the sEMG on the arm skin surface and force of the action output by the hand can be measured synchronously. The widely used time domain feature extraction methods are employed to pre-process the sEMG signals. Then the extracted EMG features are mapped to force of the hand movement by using GRNN.

The experiments are implemented to verify the effectiveness of the proposed force estimation method of hand movement. And the results show that the proposed method can realize the force estimation of hand movement with an acceptable performance under the condition that grip force is <30N and the 3D push-pull force is <20N. In grip force estimation, the result of MVA is best. In 3D force estimation, the result of MAV is also best, but compare with VAR and WA, there is no significant difference. The performance of ZC is worst both in grip estimation and 3D force estimation.

For the future work, we will research the estimation of the parameters of the kinematics and dynamics based on the sEMG for the purpose of further improving the performance of the dexterous manipulation of the EMG prostheses.

## Ethics statement

The study is exempt from ethics approval. We put up a petition in Chinese for sEMG clinical measurement to the Nanjing Tongren Hospital (http://tongren.ecaihr.com/). The relevant organization of the Nanjing Tongren Hospital consider our clinical measurement without ethical problem. All the subjects were gave written, informed consent. The permission was granted by the subject to reproduce their likeness in Figures 2, 10.

## Author contributions

CW: Project design; Experimental study; Writing paper. HZ: Data analysis. AS: Project design. BX: Data analysis.

### Conflict of interest statement

The authors declare that the research was conducted in the absence of any commercial or financial relationships that could be construed as a potential conflict of interest.
